# Regulation of dopamine neurotransmission from serotonergic neurons by ectopic expression of the dopamine D2 autoreceptor blocks levodopa-induced dyskinesia

**DOI:** 10.1186/s40478-018-0653-7

**Published:** 2019-01-15

**Authors:** Rhyomi C. Sellnow, Jordan H. Newman, Nicole Chambers, Anthony R. West, Kathy Steece-Collier, Ivette M. Sandoval, Matthew J. Benskey, Christopher Bishop, Fredric P. Manfredsson

**Affiliations:** 1 Department of Translational Science & Molecular Medicine, College of Human Medicine, Michigan State University, Grand Rapids, MI USA; 2 Cell and Molecular Biology Program, Michigan State University, East Lansing, MI USA; 3 Rosalind Franklin University, North Chicago, USA; 4 Department of Psychology, Binghamton University, Binghamton, NY USA; 5 Mercy Health Saint Mary’s, Grand Rapids, MI USA

**Keywords:** 5-HT, Serotonin, DA, Dopamine, Dyskinesia, L-DOPA, Dorsal raphe, AAV, Gene therapy

## Abstract

Levodopa-induced dyskinesias (LID) are a prevalent side effect of chronic treatment with levodopa (L-DOPA) for the motor symptoms of Parkinson’s disease (PD). It has long been hypothesized that serotonergic neurons of the dorsal raphe nucleus (DRN) are capable of L-DOPA uptake and dysregulated release of dopamine (DA), and that this “false neurotransmission” phenomenon is a main contributor to LID development. Indeed, many preclinical studies have demonstrated LID management with serotonin receptor agonist treatment, but unfortunately, promising preclinical data has not been translated in large-scale clinical trials. Importantly, while there is an abundance of convincing clinical and preclinical evidence supporting a role of maladaptive serotonergic neurotransmission in LID expression, there is no direct evidence that dysregulated DA release from serotonergic neurons impacts LID formation. In this study, we ectopically expressed the DA autoreceptor D2R_s_ (or GFP) in the DRN of 6-hydroxydopamine (6-OHDA) lesioned rats. No negative impact on the therapeutic efficacy of L-DOPA was seen with rAAV-D2R_s_ therapy. However, D2R_s_ treated animals, when subjected to a LID-inducing dose regimen of L-DOPA, remained completely resistant to LID, even at high doses. Moreover, the same subjects remained resistant to LID formation when treated with direct DA receptor agonists, suggesting D2R_s_ activity in the DRN blocked dyskinesogenic L-DOPA priming of striatal neurons. In vivo microdialysis confirmed that DA efflux in the striatum was reduced with rAAV-D2R_s_ treatment, providing explicit evidence that abnormal DA release from DRN neurons can affect LID. This is the first direct evidence of dopaminergic neurotransmission in DRN neurons and its modulation with rAAV-D2R_s_ gene therapy confirms the serotonin hypothesis in LID, demonstrating that regulation of serotonergic neurons achieved with a gene therapy approach offers a novel and potent antidyskinetic therapy.

**Electronic supplementary material:**

The online version of this article (10.1186/s40478-018-0653-7) contains supplementary material, which is available to authorized users.

## Introduction

The hallmark motor symptoms in Parkinson’s disease (PD) arise following substantial dopaminergic denervation within the striatum. Denervation results from the death of tyrosine hydroxylase (TH) expressing DA neurons of the substantia nigra pars compacta (SNc) as the disease progresses [35, 41]. The lack of proper DA signaling to the striatum creates an imbalance of the basal ganglia motor circuit, thus, causing bradykinesia, rigidity, tremor, and gait problems characteristic of PD [29]. Current treatment strategies, while not able to affect disease progression, are aimed at treating these primary motor symptoms. Since the late 1960s, L-3,4-dihydroxyphenylalanine (levodopa or L-DOPA) has been used as a catecholamine replacement therapy to alleviate motor symptoms [21]. L-DOPA remains the gold-standard pharmacological treatment for PD.

While effective initially, the therapeutic window of L-DOPA narrows with the continuous loss of SNc neurons as the disease progresses, and higher doses are required to maintain the anti-akinetic effects of L-DOPA. Moreover, chronic treatment with L-DOPA leads to the development of L-DOPA-induced dyskinesias (LID), a series of motor symptoms distinct and independent from the PD motor deficits being treated (reviewed in [3]). These symptoms, comprised of painful and disrupting movements including hyperkinesia, dystonia, and chorea, occur in a majority of PD patients, developing in up to 50% of patients within 5 years of beginning treatment, and up to 90% of patients within 10 years [1, 49].

Studies show that LID development is a multifaceted process. However, it is largely agreed upon that the intermittent oral dosing of L-DOPA results in large variations in extracellular DA. Ultimately, this pulsatile release of DA, together with the denervated state of the striatum, results in maladaptive molecular and structural changes in the DA-responsive neurons of the striatum, specifically medium spiny neurons (MSNs), leading to altered basal ganglia signaling (reviewed in [16]). Given the extreme nigrostriatal denervation at the time of diagnosis [41], the actual source of striatal DA following L-DOPA administration has been debated over the past half century. The leading hypothesis is that uptake of L-DOPA and its subsequent dysregulated metabolism to DA, and release by serotonergic 5-hydroxytryptamine (5-HT) neurons in the dorsal raphe nucleus (DRN) may be linked to dyskinesogenesis (reviewed in [23]). These neurons express aromatic L-amino acid decarboxylase (AADC) and can therefore convert L-DOPA into DA. However, DRN neurons do not express the regulatory mechanisms to monitor and control DA synthesis and release into the synapse, allowing for the unregulated release of DA into a hypersensitized striatum [46]. Additionally, serotonergic innervation of the striatum increases substantially following DA denervation, allowing the majority of L-DOPA to be metabolized and released as DA by serotonergic terminals [45, 47, 64, 65, 77]. This overwhelming exposure of the DA-depleted striatal MSNs to exogenous DA is hypothesized to be a large contributor to LID. In fact, studies in rats show that specifically lesioning the DRN [14, 24] or co-administering L-DOPA with 5-HT1 receptor agonists [8, 28, 52, 61], effectively reduces or eliminates LID.

Normal regulation of DA signaling is mediated presynaptically primarily through the DA active transporter (DAT) and the DA autoreceptor. DAT directly regulates the levels of DA in the synapse by transporting synaptic DA back into the terminal. The dopamine autoreceptor (D2R_s_) is an isoform of the D2 DA receptor (D2R_L_) missing 29 amino acids from the third intracellular loop [22]. D2R_s_ detects synaptic DA levels and regulates DA signaling in three ways, 1) by downregulating DA production through TH regulation, 2) regulation of reuptake through DAT, and 3) by directly inhibiting DA release (reviewed in [26]). Each of these modes of action are mediated through the inhibitory G_i_ alpha protein signaling pathways following D2R_s_ activation.

These canonical G-protein-coupled receptor (GPCR) signaling pathways similarly inhibit serotonergic signaling in DRN neurons through 5-HT1 autoreceptor activation [34, 57]. Previous studies using 5-HT1 agonists show promising reductions in LID. Unfortunately, these drugs can negate the anti-parkinsonian therapeutic benefits of L-DOPA animal models, and in some cases worsen PD symptoms in clinical trials [19, 36, 37, 58].

While current evidence suggests a crucial role of serotonergic input and activity in LID, direct evidence of the abnormal dopaminergic neurotransmission and dysregulated DA release is lacking. In the present study, we sought to provide unequivocal evidence for the role of serotonergic DA neurotransmission in dyskinesogenesis and examine a novel therapeutic approach of modulating this non-physiological adaptation in the parkinsonian brain. To do this, we provided serotonergic neurons with DAergic regulatory mechanisms by ectopically expressing the D2R_s_ autoreceptor in the DRN of parkinsonian 6-OHDA lesioned rats, and evaluated the effect of ectopic D2R_s_ activity on L-DOPA efficacy, LID formation, response to DA agonists, and striatal DA release.

## Materials and methods

### Adeno-associated virus production

The D2R_s_ and GFP coding sequences were cloned into AAV genomes under the control of the chicken β-actin/cytomegalovirus (CBA/CMV) promoter for ubiquitous and robust expression. AAV 2/9 was produced via triple-transfection of HEK 293 T cells with the genome and helper plasmids. Virus was recovered from cells using freeze-thaw cycles, purified using an iodixanol gradient (Optiprep Density Gradient, Sigma-Aldrich, St. Louis, MO), followed by buffer exchange and concentration using concentrator columns (Orbital Biosciences, Topsfield, MA) as described previously [6]. The viral titer was determined using digital droplet PCR (ddPCR) and normalized to 1 × 10^13^ vector genomes (vg)/ml using Balanced Salt Solution (Sigma-Aldrich, St. Louis, MO).

### Animals and surgeries

Studies were performed using adult male Fischer F344 rats (200-220 g upon arrival; Charles River, Wilmington, MA) in accordance with the guidelines of Michigan State University (AUF 06/16–093-00), Binghamton University (AUF# 779–17), and Rosalind Franklin University (AUF# A3279–01) Institutional Animal Care & Use Committees. All work was performed in accordance with the ethical standards as laid down in the 1964 Declaration of Helsinki and its later amendments or comparable ethical standards. Rats were housed two per cage prior to behavioral testing, and then separated and individually housed with environment enrichment during behavior studies for the remainder of the experiments. The animals were housed in a light-controlled (12 h light/dark cycle) and temperature-controlled (22 ± 1 °C) room, and had free access to standard lab chow and water.

All 6-OHDA and vector surgeries were performed under 2% isoflurane. After being anesthetized, animals were placed in a stereotaxic frame and were injected using a glass capillary needle fitted to a Hamilton syringe (Hamilton, Reno, NV) [5]. Three weeks following lesion surgery, animals were tested for spontaneous forepaw use (cylinder test) to estimate lesion efficacy. Vector treatment groups were normalized using forepaw deficits in order to ensure equal lesions between the treatment groups.

For lesion surgeries 5 mg/ml 6-OHDA hydrobromide (Sigma-Aldrich, St. Louis, MO) was prepared in 0.2 mg/ml ascorbic acid immediately prior to the injections. Animals received 2 μl injections of 6-OHDA into the medial forebrain bundle (MFB) (from bregma: Anterior Posterior (AP) – 4.3 mm, Medial Lateral (ML) + 1.6 mm, Dorsal Ventral (DV) -8.4 mm from skull) and the SNc (from bregma: AP -4.8 mm, ML + 1.7 mm, DV -8.0 mm from skull), for a total of 10 μg 6-OHDA per site and 20 μg per animal. The glass needle was lowered to the site and the injection started after 30 s. 6-OHDA was injected at a rate of 0.5 μl/minute. The needle was removed 2 minutes after the injection was finished and cleaned between each injection.

Vector delivery was performed 3 weeks following the 6-OHDA lesion via stereotaxic delivery [5]. A subset of animals (*N* = 7) destined for electrophysiological measures did not receive a 6-OHDA lesion. Using the same procedure as described for the lesion surgeries, animals received a single midline 2 μl injection of virus (AAV2/9-DR_s_, 1 × 10^13^ vg/ml; AAV2/9-GFP, 1 × 10^13^ vg/ml) to the DRN (from bregma: AP -7.8, ML -3.1, DV -7.5 from skull). The stereotaxic arm was positioned in a 30° lateral angle in order to avoid the cerebral aqueduct.

Parkinsonian and vector-injected animals used for in vivo microdialysis were shipped to Binghamton University 2 weeks following the vector surgeries. Following quarantine, rats were acclimated to the colony room and habituated to handling for 1 week. Rats were then tested for baseline forepaw adjusting steps. Thereafter, microdialysis cannulation surgery was performed under 2–3% isoflurane in oxygen with the tooth bar set to 5 mm below the interaural line. Five minutes before surgery and 24 h after surgery rats received an injection of Buprinex (0.03 mg/kg, i.p.). A unilateral dorsal striatal-directed cannula (CMA 12 Elite; Stockholm, Sweden) was implanted ipsilateral to lesion (from bregma AP: 1.2 mm; ML: − 2.8 mm; DV: − 3.7 mm). The cannula was fixed in place by four jeweler’s screws, jet liquid, and dental acrylic (Lang Dental, Wheeling, IL). Two weeks following cannulation surgery, rats underwent behavioral testing.

Non-lesioned rats used for electrophysiological recordings of the DRN were shipped to Rosalind Franklin University 2 weeks following the vector surgeries and housed for an additional 4–8 weeks prior to electrophysiological recordings. Burr holes (~ 1 mm in diameter) were drilled in the skull overlying the DRN. Prior to experimentation all animals were anesthetized with urethane (1.5 g/kg i.p.) and placed in a stereotaxic apparatus. Bipolar stimulating/recording electrodes were implanted in the frontal cortex and DRN on the right side using a micromanipulator (coordinates from Bregma: AP: 3.2 mm; ML: 0.8 mm lateral; DV: 4.4 mm ventral (frontal cortex) or AP: 7.8 mm; ML 3.1 mm; DV: 7.5 mm with the manipulator angled 30 degrees toward Bregma) as previously described [17].

### Abnormal involuntary movement (AIM) ratings and drug treatments

**Fig. 1:**
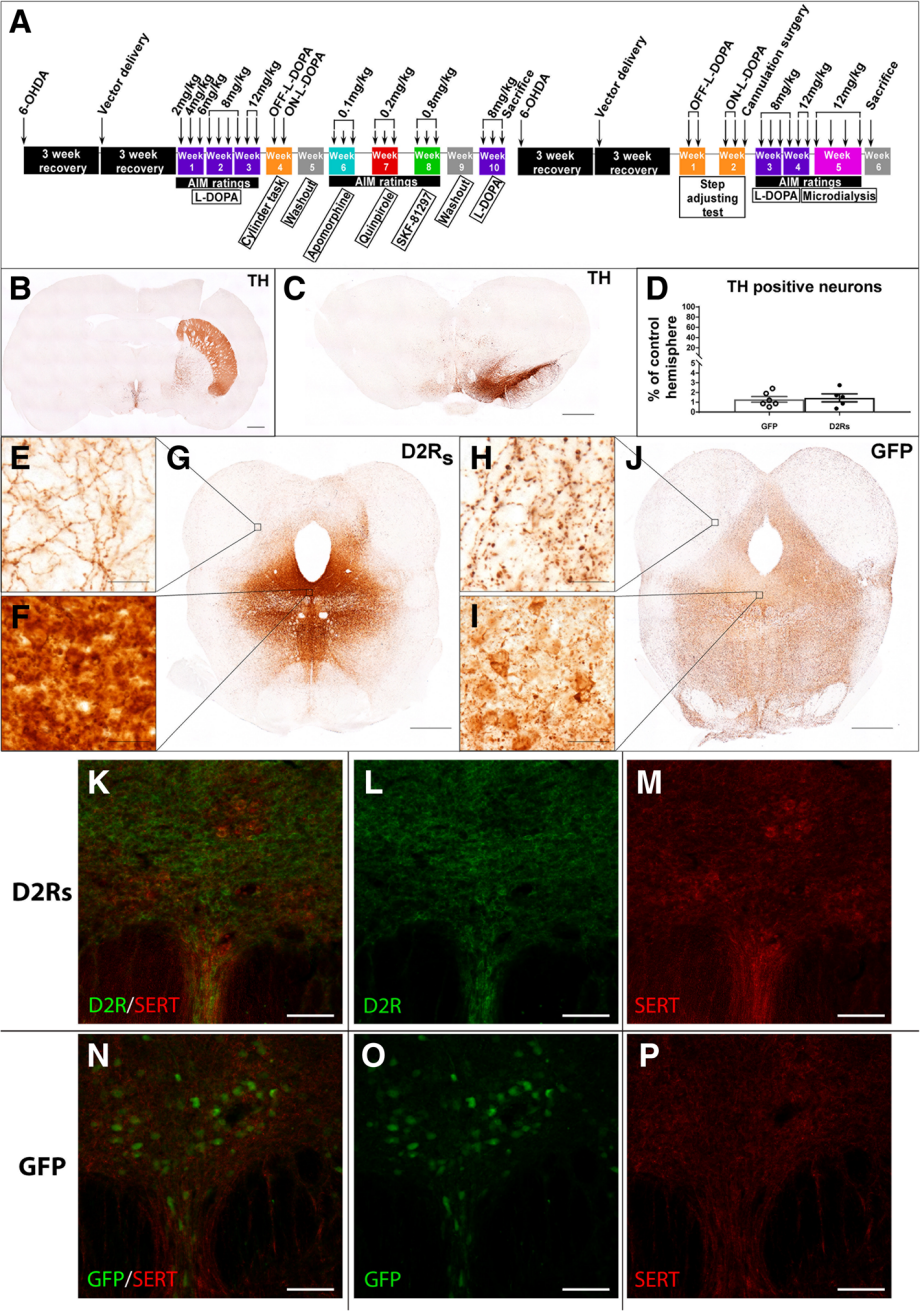
Experimental design and model validation. **a** Experimental timeline showing LID-inducing L-DOPA paradigm, motor behavior evaluations, and DA agonist treatments. AIM score ratings were taken at each injection where indicated. **b** and **c** Representative TH immunoreactivity in the striatum (**b**) and substantia nigra (**c**) showing complete loss of TH-positive neurons and projections following 6-OHDA lesions (scale bar = 1 mm). **d** Total enumeration of remaining TH neurons in the substantia nigra. **e** and **f** IHC for the D2 receptor (**e**) or GFP (**f**) in the DRN, showing successful targeting of the structure and robust expression of the transgene (scale bar = 1 mm). Cell bodies were efficiently transduced in the DRN (**e** and **f**, bottom insets, scale bar = 50um) and could be seen filling projection fibers in the peduncles (**e** and **f**, top insets, scale bar = 50um). **k**-**p** Dual labeling transgene expression and SERT in rAAV-D2R_s_ **k**-**m** and rAAV-GFP (**n**-**p**) animals. Transgene expression was visualized with D2R_s_ (**l**) or GFP (**o**) staining, and serotonin fiber and cell integrity were confirmed by staining for SERT (**m** and **p**). No adverse effects on SERT fibers were observed following vector transduction with either construct (**k** and **n**) (**k**-**p** scale bar = 100um)

Animals were allowed to recover for 3 weeks following vector injections, and to allow for peak expression of the viral transgene [63]. After this time, L-DOPA treatment and abnormal involuntary movement (AIM) scale ratings began (see time line in Fig. 1a). As described previously, the AIM rating scale can be used to evaluate the severity of LID and has been adapted for animal use [44, 71]. Briefly, AIMs are evaluated by scoring the level of dystonia of the limbs and body, hyperkinesia of the forelimbs, and orolingual movements. Each AIM is given two numerical scores—one indicating the intensity (0 = absent, 1 = mild, 2 = moderate, or 3 = severe) and frequency (0 = absent, 1 = intermittently present for < 50% of the observation period, 2 = intermittently present for > 50% of the observation period, or 3 = uninterruptable and present through the entire rating period) [50]. Each AIM is given a severity score by multiplying the intensity and frequency, and the total AIM score is a sum of all the behaviors severities. An animal is considered non-dyskinetic with a score of ≤4, as non-dyskinetic parkinsonian rats can display low level AIMs from exhibiting normal chewing behavior and a mild parkinsonian dystonic posture [78].

Animals received subcutaneous injections of L-DOPA/benserazide (Sigma-Aldrich, St. Louis, MO) three times per week and were rated using the AIM scale in 25-min intervals post-injection until all LID behavior had subsided. L-DOPA doses ranged between 2 mg/kg-12 mg/kg (Fig. 1a). Benserazide doses (12 mg/kg) remained constant for all L-DOPA injections. The same injection and rating paradigm was used for AIM evaluations with the non-selective DA agonist apomorphine (0.1 mg/kg, R&D Systems, Minneapolis, MN), the D2/D3 receptor agonist quinpirole (0.2 mg/kg, Sigma-Aldrich, St. Louis, MO) and the D1 receptor agonist SKF-81297 (0.8 mg/kg, Sigma-Aldrich, St. Louis, MO). DA agonist doses were selected based on doses known to induce AIMs in parkinsonian rats [9, 42]. Peak AIM scores of DA agonists were determined based off the highest average AIM scores of control animals during the rating period.

### Parkinsonian motor evaluation

To assess whether D2R_s_ viral therapy affects the anti-parkinsonian properties of L-DOPA therapy, we evaluated parkinsonian motor behavior on and off L-DOPA using the cylinder task and the forepaw adjusting steps (FAS) test. Rats with significant lesions perform poorly on both these tests, with impairment to the forepaw contralateral to the lesion that is alleviated with L-DOPA treatment [18, 68]. The cylinder task was conducted as previously reported [48]. Animals were placed in a clear Plexiglas cylinder on top of a light box for 5 to 7 minutes while being recorded. Each animal was rated by counting ~ 20 weight-bearing forepaw placements on the cylinder (contralateral to the lesion, ipsilateral to the lesion, both) to determine the percentage use of the forepaw contralateral to the lesion, which is derived by dividing the sum of contralateral touches and half of both forepaw touches by the total forepaw touches, and multiplying this number by 100. Trials were performed following the initial L-DOPA treatment (AIM evaluation) period, and tested either off L-DOPA or, on the following day, 50 min after receiving a 6 mg/kg L-DOPA injection (12 mg/kg benserazide).

The FAS test was performed as described previously [52]. Briefly, rats were restrained by an experimenter so that only one forepaw was free to touch the counter. Rats were then dragged laterally along a 90 cm distance over 10 s while a trained rater blind to the experimental condition counted the number of steps. Data are represented as forehand percent intact, which are derived by taking the number of steps taken by the contralateral forehand and dividing it by the ipsilateral forehand, and then multiplying this number by 100. The test was performed over 2 days either off L-DOPA or 60 min following an 8 mg/kg or 12 mg/kg L-DOPA injection.

### Tissue collection

Two hours following the final L-DOPA administration, animals from the AIM experimentation were sacrificed via sodium pentobarbital overdose and intracardially perfused with Tyrode’s solution (137 mM sodium chloride, 1.8 mM calcium chloride dihydrate, 0.32 mM sodium phosphate monobasic dihydrate, 5.5 mM glucose, 11.9 mM sodium bicarbonate, 2.7 mM potassium chloride). Brains were rapidly removed and coronally hemisected, with the rostral portion of the left and right striatum dissected out and flash frozen in liquid nitrogen for biochemical analysis. The caudal portion of the brain was postfixed for 72 h in 4% paraformaldehyde (PFA) in phosphate-buffered saline and then cryoprotected by saturation in 30% sucrose. Brains were frozen and sectioned coronally at 40 μm thickness using a sliding microtome into free floating sections and stored in cryoprotectant (30% ethylene glycol, 0.8 mM sucrose in 0.5× tris-buffered saline) until further use.

### Immunohistochemistry

A 1:6 series of free-floating tissue was stained immunohistochemically for TH (MAB318, MilliporeSigma, Burlington, MA), D2R (AB5084P, MilliporeSigma, Burlington, MA), GFP (AB290, Abcam, Cambridge, United Kingdom), IBA1 (019–19,741, Wako Life Sciences, Richmond, VA), or 5-HT (NT-102, Protos Biotech, New York, NY) using methods previously reported [7]. Sections were washed in 1× Tris-buffered saline (TBS) with .25% Triton x-100, incubated in 0.3% H_2_O_2_ for 30 min, and rinsed and blocked in 10% normal goat serum for 2 h. Tissue was incubated in primary antibody (TH 1:4000, D2R 1:1000, GFP 1:20,000, IBA1 1:4000, 5-HT 1:10,000) overnight at room temperature. After washing, tissue was incubated in secondary antibody (biotinylated horse anti-mouse IgG 1:500, BA-2001; Vector Laboratories, Burlingame, CA; biotinylated goat anti-rabbit IgG 1:500, AP132B, Millipore-Sigma, Burlington, MA) followed by the Vectastain ABC kit (Vector Laboratories, Burlingame, CA). Tissue staining was developed with 0.5 mg/ml 3,3′ diaminobenzidine (DAB, Sigma-Aldrich, St. Louis, MO) and 0.03% H_2_O_2_. Sections were mounted on glass slides, dehydrated, and coverslipped with Cytoseal (ThermoFisher, Waltham, MA).

Tissue for immunofluorescence dual labeling of D2R_s_ or GFP with SERT (340–004, Synaptic Systems, Goettingen, Germany) were washed with 1× TBS with 0.25% Triton x-100, blocked in 10% normal goat serum for 2 h, and probed with primary antibody overnight (D2R_s_ 1:1000, GFP 1:20,000, SERT 1:300). Tissue was incubated with secondary antibody (A11008 1:500, A11076 1:500; ThermoFischer, Waltham, MA) in the dark for 2 hours, and washed in TBS before being mounted and coverslipped with Vectashield Hardset Antifade Mounting Medium (Vector Laboratories, Burlingame, CA).

Images were taken on a Nikon Eclipse 90i microscope with a QICAM fast 1394 camera (fluorescence; QImaging, Surrey, British Columbia, Canada) or a Nikon D-1 camera (brightfield microscopy; Nikon, Tokyo, Japan). The figures were made using Photoshop 7.0 (Adobe, San Jose, CA) with the brightness, sharpness, and saturation adjusted only as needed to best represent the staining as it is viewed directly under the microscope.

### In vivo microdialysis

As outlined above, a separate cohort of parkinsonian rats treated with GFP or D2R_s_ were utilized for in vivo microdialysis. The night before the procedure, striatal probes (CMA 12 Elite; membrane length = 3 mm; 20,000 Da; Stockholm, Sweden) were inserted into the guide cannula so that they extended from bregma DV: − 3.7 to − 6.7 mm within the dorsal striatum. Rats underwent microdialysis at least 2 days following the last L-DOPA administration. During microdialysis, rats received intrastriatal infusion of filtered artificial cerebrospinal fluid (aCSF) (128 mM NaCl, 2.5 mM KCl, 1.3 mM CaCl_2_, 2.1 mM MgCl_2_, 0.9 mM NaH_2_PO_4_, 2.0 mM Na_2_HPO_4_, and 1.0 mM glucose, pH 7.4). Dialysate samples were collected every 20 min. Briefly, rats were habituated to microdialysis for 1 h. Fifty minutes into the procedure, rats received a subcutaneous injection of L-DOPA vehicle, which consisted of 0.9% NaCl, and 0.1% ascorbate. Rats then underwent baseline testing for 1 hour to determine baseline levels of monoamines prior to L-DOPA treatment. After that a new collection tube was used and 10 minutes later rats received an injection of L-DOPA (12 mg/kg + 12 mg/kg Benserazide, s.c.). Samples were taken every 20 min for 3 h. Following the procedure, rats were removed from the microdialysis bowl and striatal probes were replaced with a dummy probe. At least 2 days after microdialysis, rats were sacrificed via rapid decapitation, the anterior striatum was taken for verification of cannula placement, the posterior striatum was taken for HPLC, and the hindbrain was placed in 4% PFA for 3 days before being placed in 30% sucrose in phosphate-buffered saline (PBS). Brains were shipped on ice in a 50 mL conical containing 30% sucrose in 0.1 M PBS to MSU.

### High-performance liquid chromatography for monoamine tissue analysis

Striatal tissue and in vivo microdialysis samples were analyzed using HPLC. Reverse-phase HPLC was performed on striatal tissue samples as previously described [38, 52]. Briefly, tissue samples were homogenized in ice-cold perchloric acid (0.1 M) with 1% ethanol and 0.02% ethylenediaminetetraacetic acid (EDTA). Homogenate was spun at 4 °C for 45 min at 14,000 g. Supernatant was removed and, using an ESA solvent delivery system (Model 542; Chelmsford, MA, USA) ESA autoinjector (Model 582), analyzed for levels of norepinephrine, 3,4-dihydroxyphenylacetic acid (DOPAC), DA, 5-hydroxyindoleacetic acid (5-HIAA), and 5-HT. Monoamines and metabolites were detected as a generated current as a function of time by EZCHROM ELITE software via a Scientific Software, Inc. (SS240x) Module. Data are displayed as peaks for monoamines and metabolites, which are compared to a standard curve made from monoamine and metabolite samples of known concentrations ranging from 1e-6 to 1e-9. Values were then normalized to tissue weight and lesion deficits are reported as percent depletion, which is equal to 100 (1 – M Lesion/ *M* Intact).

Dialysate samples were analyzed via reverse-phase HPLC on an Eicom HTEC-500 System (Amuza Inc., San Diego, CA). Briefly, 10 μL of each dialysate sample was analyzed for NE, DA, and 5-HT using an Eicompak CAX column maintained at 35 °C with a flow rate of 250 μL/min. Mobile phase (75 mM Ammonium acetate, 9.36 mM acetic acid, 1.33 mM EDTA, 0.94 mM Methanol, 50 mM sodium sulfate). Samples were compared to known concentrations of monoamines (100, 10, 1, 0.1, and 0.05 ng/μL dissolved in a potassium phosphate buffer (0.1 mM potassium phosphate monobasic, 0.1 mM ethylenediaminetetraacetic acid, 0.02 mM phosphoric acid), resulting in a final value of monoamine in ng/μL. 

### Total enumeration of TH+ and 5-HT+ neurons

Lesion severity was determined using total enumeration of TH-positive neurons in three representative sections within the SNc identified by the presence and proximity to the medial terminal nucleus (MTN) of the accessory optic tract at levels equivalent to − 5.04 mm, − 5.28 mm and − 5.52 mm relative to bregma according to our previously validated method [30]. Briefly, the intact and lesion SNc were quantified for all TH immunoreactive cells using a 20× objective and MicroBrightfield StereoInvestigator software (MicroBrightfield Bioscience, Williston, VT). The total number of TH cells on the intact and lesioned hemispheres were averaged, and lesion efficacy was derived by dividing the lesioned hemisphere average by the intact hemisphere average and multiplying that value by 100.

Total number of 5-HT positive neurons in the DRN were also quantified with total enumeration [30]. Three sections of the DRN were quantified for all 5-HT immunoreactive cells under a 20× objective using MicroBrightfield StereoInvestigator (MicroBrightfield Bioscience, Williston, VT). The total number of from all three sections per animal were summed to give a total number of 5-HT neurons.

### Electrophysiology

Recording microelectrodes were manufactured from 2.0 mm OD borosilicate glass capillary tubing and filled with sodium chloride (2 M) solution. Electrode impedance was 5-15 MΩ. The signal to noise ratio for all recordings was > 4:1. The level of urethane anesthesia was periodically verified via the hind limb compression reflex and maintained using supplemental administration as previously described [59, 66]. Temperature was monitored using a rectal probe and maintained at 37C° using a heating pad (Vl-20F, Fintronics Inc., Orange, CT). Electrical stimuli (duration = 500 μs, intensity = 1000 μA) were generated using a Grass stimulator and delivered in single pulses (0.5 Hz) while searching for cells [59]. Once isolated, recordings consisted of basal (pre-drug), saline vehicle, and drug-treatment-(see below) induced changes in spike activity recorded in a series of 3 min duration epochs.

All compounds and physiological 0.9% saline were prepared daily and administered intravenously (i.v.) through the lateral tail vein to enable rapid examination of potential acute effects of vehicle or drug on DRN neuronal activity. The selective 5-HT1A agonist 8-OH-DPAT (5 μg/kg, i.v.), the selective 5-HT1A antagonist WAY100635 (100 μg/kg, i.v.), and the D2R agonist Quinpirole (500 μg/kg, i.v.) were dissolved in vehicle and administered systemically to either BFP or D2R_s_ rats. DRN 5-HT neuron activity was recorded prior to and immediately following drug administration as described above.

### Statistical analysis

Statistical analysis was performed using Statview (version 5.0) or in SPSS version 23 with α set to 0.05. All graphs were created in GraphPad Prism version 7.0 (GraphPad Software, La Jolla, CA) or Excel (Microsoft, Redmond, WA). Lesion status was evaluated using unpaired, one-tailed t-tests. AIMs were evaluated using a non-parametric Mann-Whitney U test, with *p* ≤ 0.05 being considered statistically significant. Bonferroni post-hoc tests were employed when significant main effects were detected. Cylinder and FAS data for forehand and backhand stepping were submitted to a mixed model ANOVA with within-subjects factors of treatment (2: Baseline, L-DOPA) and between-subjects factors of vector (GFP, D2R). Overall percent intact values for FAS were determined by taking the overall number of right paw steps divided by the number of left paw steps and multiplying the quotient by 100. Similarly, overall percent intact values were analyzed via a repeated-measures ANOVA with within-subjects factor of treatment and between subjects factor of vector. Monoamine content (as determined by HPLC) was submitted to a mixed-model ANOVA with within-subjects factor of treatment (2: Vehicle, L-DOPA) and between-subjects factor of vector. Fisher’s least significant difference (LSD) post-hocs and planned paired-samples t-tests were employed as appropriate to clarify significant effects. Additionally, independent-samples t-tests were employed to reveal effects of vector on the timing of DA, NE, and 5-HT efflux. HPLC values for striatal tissue were submitted to a mixed-model ANOVA with within-subjects factor of side and between-subjects factor of vector. Subsequently, since DA depletion did not vary as a function of vector, values for each monoamine for each side were collapsed across treatments and compared via paired-samples t-tests. For electrophysiology experiments, the difference between the spontaneous and evoked electrophysiological activity of identified DRN-5-HT neurons across groups was determined and served as the dependent variable for our analyses. A two-way repeated measures ANOVA (GFP vs. gene therapy (ectopic expression of the DA D2 AR in 5-HT DR neurons)) × 2 (vehicle vs. drug treatment) with α set to 0.05 and all “n’s” adequately powered for electrophysiological studies was conducted using Sigma Stat software (San Jose, CA), and the potential two-way interaction effect was examined to determine how treatment effects differ as a function of drug treatment or gene therapy [59].

## Results

### Validation of lesion and transgene expression

In order to assess if exogenous expression of D2R_s_ in the DRN could inhibit LID development or decrease LID severity, adult Fischer rats were rendered parkinsonian with 6-OHDA delivered to the SNc and MFB. Because LID is dependent on the severity of the lesion [76] we validated post mortem that sufficient nigrostriatal denervation was achieved. Immunohistochemistry of the striatum (Fig. 1b) and the SNc (Fig. 1c) showed a near complete ablation of TH immunoreactivity with no difference in the number of SNc DA neurons between groups (Fig. 1 d; GFP = 1.29% + 0.29% remaining; D2R_s_ = 1.45% + 0.41% remaining; t_(9)_ = 0.31, *p* > 0.05). Similarly, HPLC analysis of DA and DOPAC levels from striatal tissue from rats employed in the microdialysis experiment confirmed that all animals displayed an almost near complete reduction in striatal DA levels in the lesioned hemisphere as compared to the intact hemisphere (DOPAC = 18.11 ± 6.68% of intact hemisphere, DA = 3.48 ± 1.36% of intact hemisphere) (Additional file 1: Figure S1). There was no difference in striatal DA depletion between groups (DOPAC t_(13)_ = 0.73, *p* > 0.05, DA t_(13)_ = 17.21, *p* > 0.05).

After a three-week recovery period, rAAV 2/9 expressing either D2R_s_ or GFP was delivered by stereotaxic injection into the DRN. Following sacrifice, transduction was confirmed with immunohistochemistry (IHC) of D2R_s_ or GFP (Fig. 1 e-j, Additional file 2: Figure S2). Significant transgene expression was observed in the soma (D2R_s_ Fig. 1f, g; GFP Fig. 1i, j) of the DRN as well as in DRN efferent projections (D2R_s_ Fig. 1e; GFP Fig. 1h, Additional file 2: Figure S2). The two transgenes exhibited a slightly different subcellular expression pattern where more GFP expression was seen in projections as compared to D2R_s_ expression (Additional file 2: Figure S2). It is unclear if this is due to increased 5-HT innervation in dyskinetic (i.e. GFP treated) animals [45], or due to a different distribution pattern specific to the transgenes. The latter is to be expected as GFP is a soluble protein and typically fills the entire neuron. Transduction expression was observed throughout the brain, however, all transgene immunoreactivity anterior to the DR was localized to projections and not cell bodies (Additional file 2: Figure S2c, d, g, h). An evaluation of Iba1 immunoreactivity indicated that transduction of either vector did not result in inflammation, as the only increase in Iba1 was seen at the injection site itself (Additional file 2: Figure S2e, i) and this response did not differ between groups. Vector transduction and transgene expression of either construct did not adversely affect SERT expression in the DRN (Fig. 1k-p). Additionally, total enumeration of 5-HT positive neurons in the DRN showed no effect of vector expression on the number of cells (Additional file 2: Figure S2L). Four animals (rAAV-D2R_s_: *n* = 2, rAAV-GFP: n = 2) that lacked sufficient vector expression in the DRN were removed from the analysis, leaving a total of *n* = 15 rats included in the analysis (rAAV-D2R_s_: *n* = 7, rAAV-GFP: *n* = 8).

### D2R_s_ delivery to the dorsal raphe eliminates LID

**Fig. 2:**
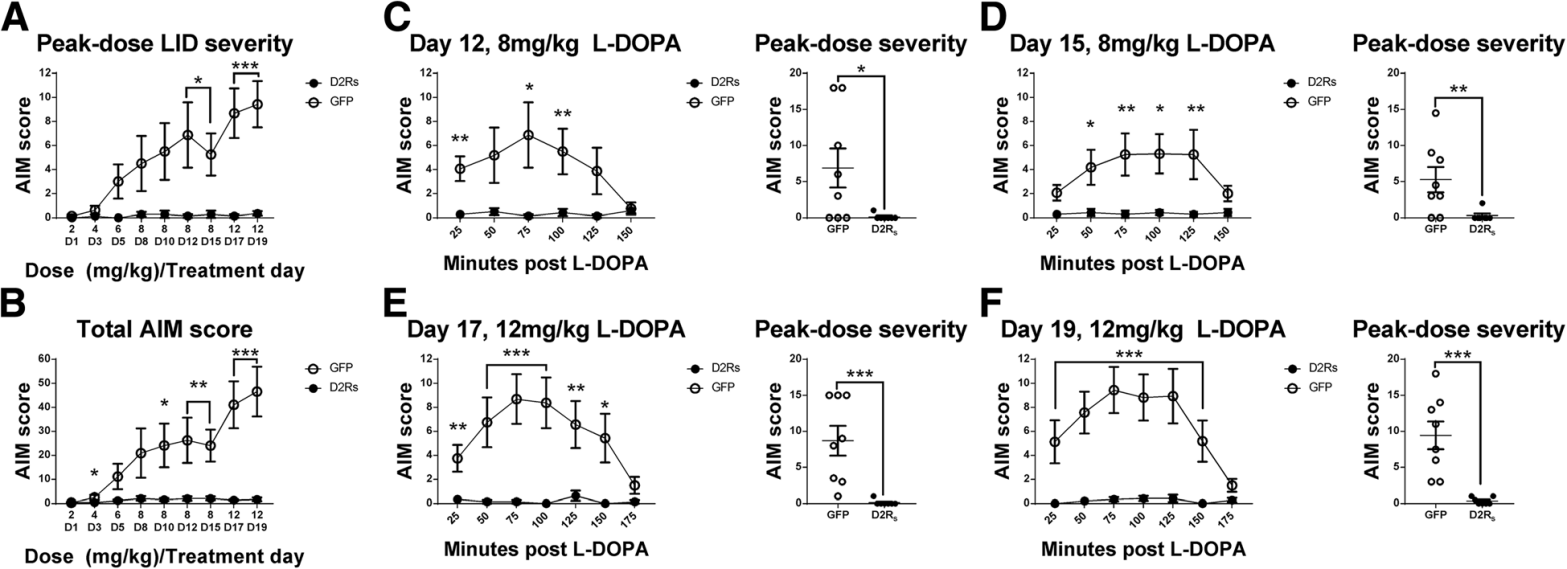
DRN D2R_s_ expression blocks LID development. **a** rAAV-D2R_s_-injected animals did not develop LID over the course of 19 days of treatment with increasing doses of L-DOPA, where rAAV-GFP controls developed AIMs. **b** The total AIM score for each rating session was significantly different between groups starting on treatment day 8 with 8 m/kg L-DOPA. D2R_s_ animals remained LID-. **c**-**f** AIM scores from days 12, 15, 17, and 19 showing LID severity in 25 min intervals. GFP animals displayed a typical dyskinetic response to chronic L-DOPA treatment, with increasing AIM severity seen at higher doses. The peak-dose severity (AIM score at 75 min-post L-DOPA injection) was significantly higher in GFP animals than D2R_s_ animals in the last 4 days of the L-DOPA paradigm. (* = *p* ≤ 0.05, ** = *p* ≤ 0.01, *** = *p* ≤ 0.001)

After a 4-week recovery period to allow for optimal transgene expression [63], animals were treated with L-DOPA and rated for AIMs (see Fig. 1a for experimental timeline). With L-DOPA, rAAV-D2R_s_ treated animals did not show significant LID at the typical peak-dose time point (75 min post L-DOPA delivery) LID (defined as an AIM score ≥ 4) [78] at any dose level (2 mg/kg AIMs = 0 ± 0, 4 mg/kg AIMs = 0.14 ± 0.14, 6 mg/kg AIMs = 0 ± 0, 8 mg/kg day 8 AIMs = 0.29 ± 0.18, 8 mg/kg day 10 AIMs = 0.29 ± 0.29, 8 mg/kg day 12 AIMs = 0.14 ± 0.14, 8 mg/kg day 15 AIMs = 0.29 ± 0.29, 12 mg/kg day 17 AIMs = 0.14 ± 0.14, 12 mg/kg day 19 AIMs = 0.36 ± 0.18) (Fig. 2a, Additional file 3: Figure S3). rAAV-GFP controls began to show mild-to-moderate peak-dose AIMS with a moderate L-DOPA dose (6 mg/kg peak dose AIMs = 3 ± 1.43), which increased to more significant levels of severity with higher doses of L-DOPA (8 mg/kg peak dose AIMs: day 8 = 4.5 ± 2.29, day 10 = 5.5 ± 2.36, day 12 = 6.88 ± 2.72, day 15 = 5.25 ± 1.76; 12 mg/kg peak dose AIMs: day 17 = 8.69 ± 2.06, day 19 = 9.44 ± 1.93) (Fig. 2a, Additional file 3: Figure S3). When compared to rAAV-D2R_s_ subjects, rAAV-GFP animals showed significantly higher total peak dose AIM scores per session starting with 8 mg/kg doses (day 12 rAAV-D2R_s_ (Md = 0), rAAV-GFP (Md = 4), U = 12, *p* < 0.05; day 15 rAAV-D2R_s_ (Md = 0), rAAV-GFP (Md = 4.5), U = 12, p < 0.05; Mann-Whitney U test) (Fig. 2b). This difference was maintained with the high dose of L-DOPA (12 mg/kg day 17 rAAV-D2R_s_ (Md = 0), rAAV-GFP (Md = 8.5), U = 0.5, *p* < 0.001; day 19 rAAV-D2R_s_ (Md = 0), rAAV-GFP (Md = 9.25), U = 0, p < 0.001) (Fig. 2c-f, Additional file 3: Figure S3). Taken together, these data show that D2R_s_ expression in the DRN completely blocks the development of LID in parkinsonian rats, even with administration of high L-DOPA doses.

### D2R_s_ does not affect parkinsonian motor behavior

**Fig. 3:**
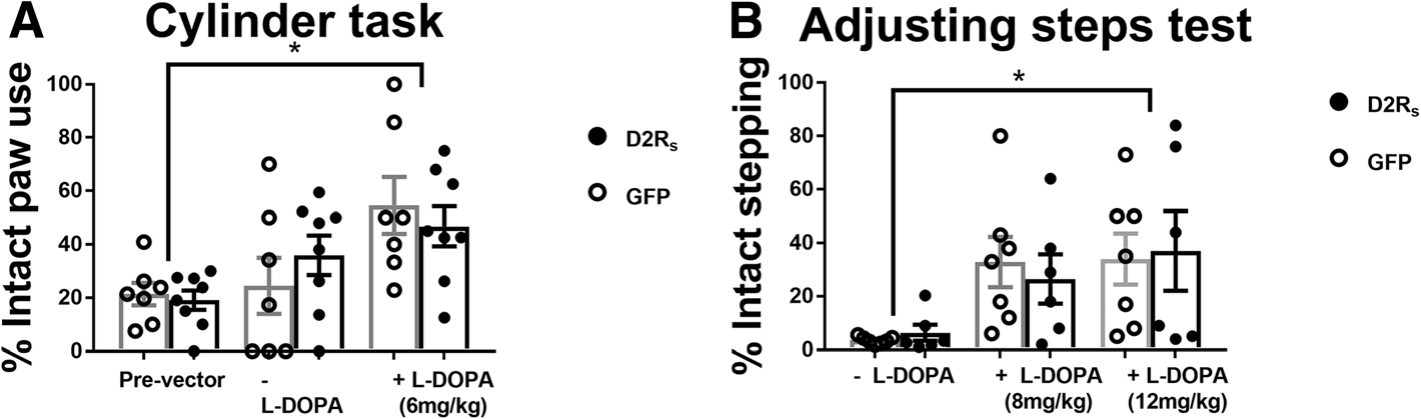
rAAV-D2R_s_ does not impact L-DOPA efficacy. **a** Cylinder task was performed 3 weeks post-lesion (pre-vector), off L-DOPA (post-vector, post-L-DOPA paradigm) and on L-DOPA (6 mg/kg, 50 min post injection). Both vector groups showed significant impairment following lesion and vector delivery, which was recovered with L-DOPA treatment. There were no significant differences between vector groups. **b** A second cohort received the same lesions and vector deliveries and motor function was evaluated using the adjusting steps tests. While all animals in both groups showed significant impairment on the test without L-DOPA, motor function was restored while on drug (8 mg/kg and 12 mg/kg). There were no differences between vector groups. * = *p* ≤ 0.01

To assess if rAAV-D2R_s_ treatment alters the anti-akinetic properties of L-DOPA, we examined motor behavior using the cylinder task (Fig. 3a). There were no significant differences between the rAAV treatment groups without L-DOPA (F_(1,13)_ = 0.008, *p* > 0.05). Pre-vector scores for both groups and post-vector scores for rAAV-GFP showed a marked decrease from normal contralateral forepaw use, indicating significant impairment. rAAV-D2R_s_ animals post-vector showed a trend towards more balanced forepaw use, but the differences were not significant. Both groups showed a significant increase from baseline increase towards balanced contralateral forepaw use while on L-DOPA (6 mg/kg) (F_(2,26)_ = 7.11, *p* < 0.01). No significant differences were seen in impairment or improvement between vector treatment groups (F_(2,26)_ = 0.72, *p* > 0.05). A second separate group of animals (microdialysis cohort) which were treated identical (lesion, vector delivery, L-DOPA paradigm) to the initial cohort of animals, underwent the adjusting steps test, both off and on (8-12 mg/kg) L-DOPA. Both vector treatment groups showed significantly impaired adjusting steps without L-DOPA (GFP baseline = 3.57% ± 0.49% intact stepping; D2R_s_ baseline = 6.33% ± 3.02% intact stepping; t_(11)_ = 0.98, p > 0.05), however, this deficit was rescued with the administration of both doses of L-DOPA (Fig. 3b; F_(2,22)_ = 9, p < 0.01). As with the cylinder task, no differences in impairment nor improvement while on L-DOPA were seen between groups (F_(2,22)_ = 0.24, p > 0.05). Together, this suggests that ectopic D2R_s_ expression in the DRN does not interfere with the anti-parkinsonian motor benefits of L-DOPA.

### Dopamine receptor agonists do not induce significant AIMs in L-DOPA-primed rAAV-D2R_s_ rats

**Fig. 4:**
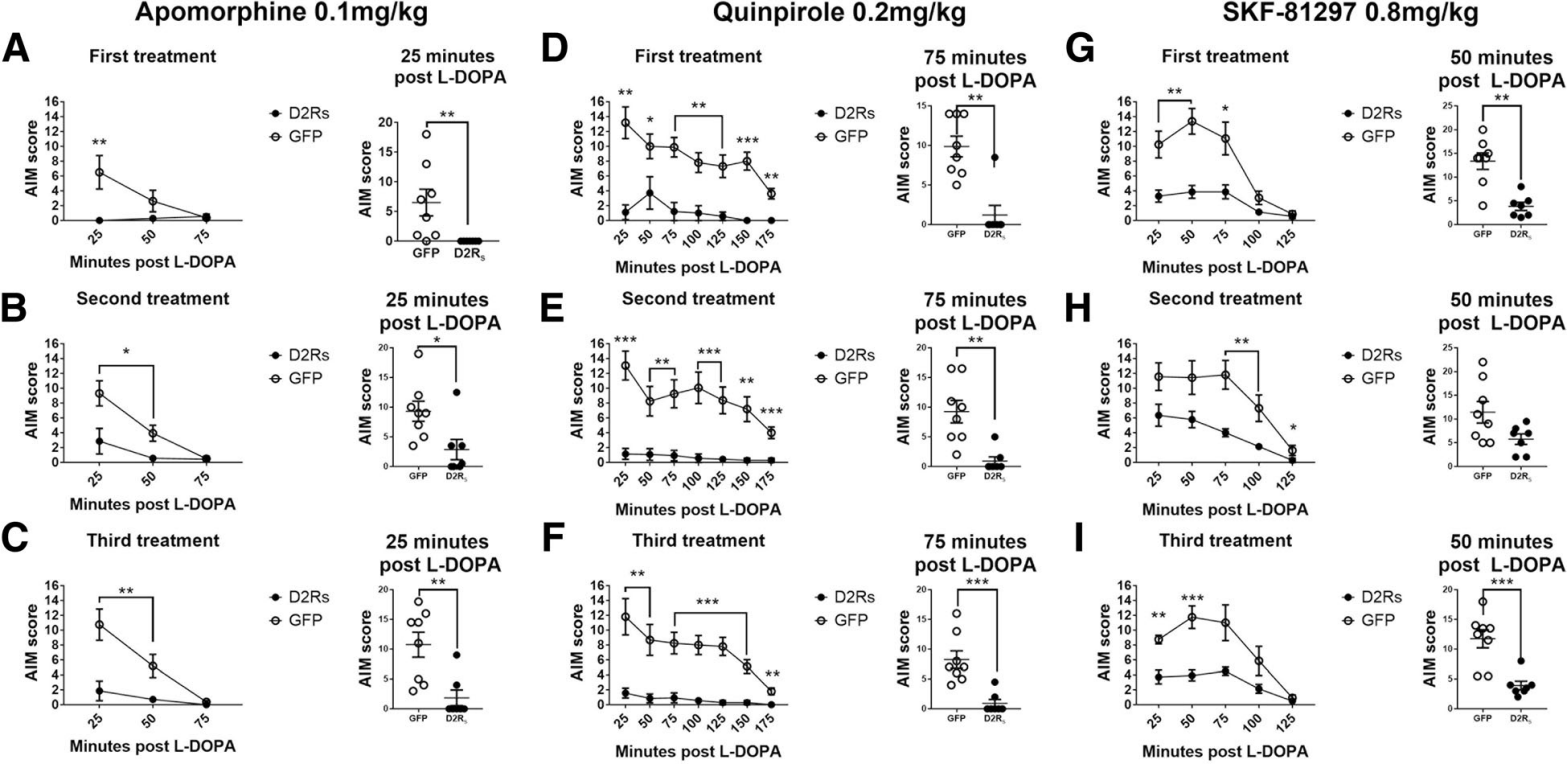
D2R_s_-injected animals do not develop severe AIMs with DA agonist treatment. Animals were treated three times each with apomorphine (**a**-**c**) quinpirole (**d**-**f**) or SKF-81297 (**g**-**i**). **a**-**c** rAAV-D2R_s_-injected animals remained AIM resistant with 0.1 m/kg pan-DA agonist apomorphine treatment, while rAAV-GFP animals continued to exhibit dyskinetic behaviors. **d**-**f** 0.2 mg/kg quinpirole (D2 agonist) did not elicit AIMs in rAAV-D2R_s_ animals, where rAAV-GFP animals continued to exhibit moderate to severe AIMs. **g**-**i** rAAV-D2R_s_ began to show mild-to-moderate AIMs with 0.8 mg/kg of the D1 agonist SKF-81297 treatments, but remained significantly less severe than their rAAV-GFP counterparts. (* = *p* ≤ 0.05, ** = *p* ≤ 0.01, *** = *p* ≤ 0.001)

Next, we examined whether dopamine agonists could induce AIMs in the rAAV-D2R_s_ treated rats that had remained resistant to LID after the L-DOPA dosing paradigm. Animals received three repeated doses each of a non-selective DA agonist (apomorphine, 0.1 mg/kg), a D2/3-specific receptor agonist (quinpirole, 0.2 mg/kg), and a D1-specific receptor agonist (SKF-81297, 0.8 mg/kg) and were evaluated for AIM severity (*see* timeline in Fig. 1a). These DA agonists can induce AIMs in both L-DOPA-primed and unprimed parkinsonian animals [11, 12, 20]. We hypothesized that directly activating the DA receptors with an agonist would bypass any protective effects of the rAAV-D2R_s_ treatment in normalizing aberrant DA release, as these agonists do not require processing and release by DAergic or serotonergic terminals, and therefore would not be affected by exogenous regulatory mechanisms. They also allowed us to compare DA receptor supersensitivity status between treatment groups. Interestingly, rAAV-D2R_s_ animals challenged with both apomorphine and quinpirole did not show significant peak AIMs (rAAV-D2R_s_ apomorphine third treatment 25 min AIMS = 1.86 ± 1.32; quinpirole third treatment 25 min AIMs = − 1.57 ± 0.66), while rAAV-GFP animals continued to express moderate-to-severe AIM behaviors (rAAV-GFP apomorphine third treatment 25 min AIMS = 10.75 ± 2.10; quinpirole third treatment 25 min AIMs = − 11.81 ± 2.45) (Fig. 4a-f). rAAV-D2R_s_ animals exhibited significantly lower peak-dose AIMs with both apomorphine and quinpirole treatment compared to rAAV-GFP animals (apomorphine third treatment 25 min AIMs rAAV-D2R_s_ (Md = 0), rAAV-GFP (Md = 12.75), U = 4.5, *p* < 0.01; quinpirole third treatment 25 min AIMs rAAV-D2R_s_ (Md = 1.5), rAAV-GFP (Md = 13), U = 3.5, p < 0.01). Treatment with SKF-81297 did induce mild-to-moderate AIM scores in rAAV-D2R_s_ treated animals (third treatment 50 min AIMs = 3.92 ± 0.73), but these scores remained significantly less severe than their control counterparts (third treatment 50 min AIMs rAAV-D2R_s_ (Md = 3.5), rAAV-GFP (Md = 13), U = 2, *p* < 0.001) (Fig. 3g-i).

### D2R_s_ expression in the dorsal raphe reduces striatal dopamine efflux following L-DOPA delivery

**Fig. 5:**
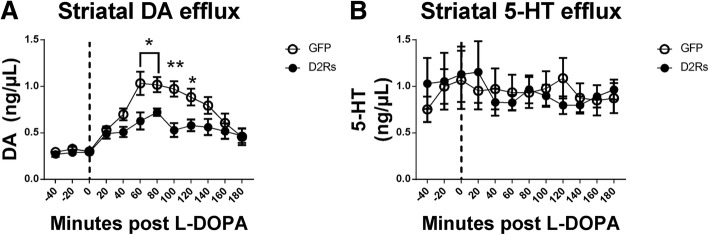
DRN D2R_s_ reduced striatal efflux of DA. **a** and **b** In vivo microdialysis of rAAV-D2R_s_ and rAAV-GFP animals in twenty-minute intervals following L-DOPA injection (12 mg/kg + 12 mg/kg Benserazide, s.c.). **a** rAAV-D2R_s_ animals showed significantly decreased DA efflux in the striatum 60–120 min following injection. **b** No changes in serotonin efflux in the striatum between vector groups was observed following L-DOPA injection. (* = *p* ≤ 0.05, ** = *p* ≤ 0.01, *** = *p* ≤ 0.001)

In order to determine if ectopic D2R_s_ expression in the DRN was inhibiting LID by moderating DA release from serotonergic neurons, we generated a second cohort of animals in order to perform in vivo microdialysis (rAAV-D2R_s_ *n* = 6, rAAV-GFP *n* = 7). Animals were lesioned and received vector in an identical manner to the first cohort, and subsequently treated with L-DOPA to establish LID. In order to determine differences between vector groups in the absence of L-DOPA, striatal dialysate was analyzed via HPLC and data for monoamine content were examined using a 2 (vector) × 2 (treatment) mixed-model ANOVA. Overall, DA values were dependent upon treatment, F_(1,11)_ = 124.35, *p* < 0.05, and vector, F_(1,11)_ = 7.39, *p* < 0.05. Planned pairwise comparisons revealed that L-DOPA treatment increased striatal DA efflux in both groups. However, rats treated with the D2R_s_ viral vector had lower levels of DA efflux than did rats treated with the GFP vector (*p* < 0.05) (Fig. 5a). Finally, there was a vector by treatment interaction, F_(1,11)_ = 6.66, *p* < 0.05, such that rats with the D2R_s_ vector had lower levels of DA efflux than rats with the GFP vector, but only after L-DOPA treatment. Striatal NE efflux was also dependent upon treatment, F_(1,11)_ = 52.10, *p* < 0.05. There was no effects of vector or treatment on striatal 5-HT efflux (Fig. 5b; F_(11,121)_ = 0.867, *p* > .05). DA values for each time point were also submitted to paired-samples t-tests in order to examine the effect of vector on DA efflux at each time point during microdialysis. There were significant differences between vector groups 60 (t_(5)_ = 3.42, *p* < 0.05), 80 (t_(5)_ = 2.77, *p* < 0.05), 100 (t_(5)_ = 4.68, *p* < 0.01), and 120 (t_(5)_ = 2.59, *p* < 0.05) minutes after L-DOPA administration, showing that rats with the GFP vector had elevated striatal DA efflux as compared to the rats with the D2R_s_ vector. This is the first direct evidence showing that mishandled DA by DRN neurons can be regulated exogenously, and this regulation reduces DA release in the striatum, thus suppressing LID.

### D2R_s_ expression inhibits 5-HT neuron activity

**Fig. 6:**
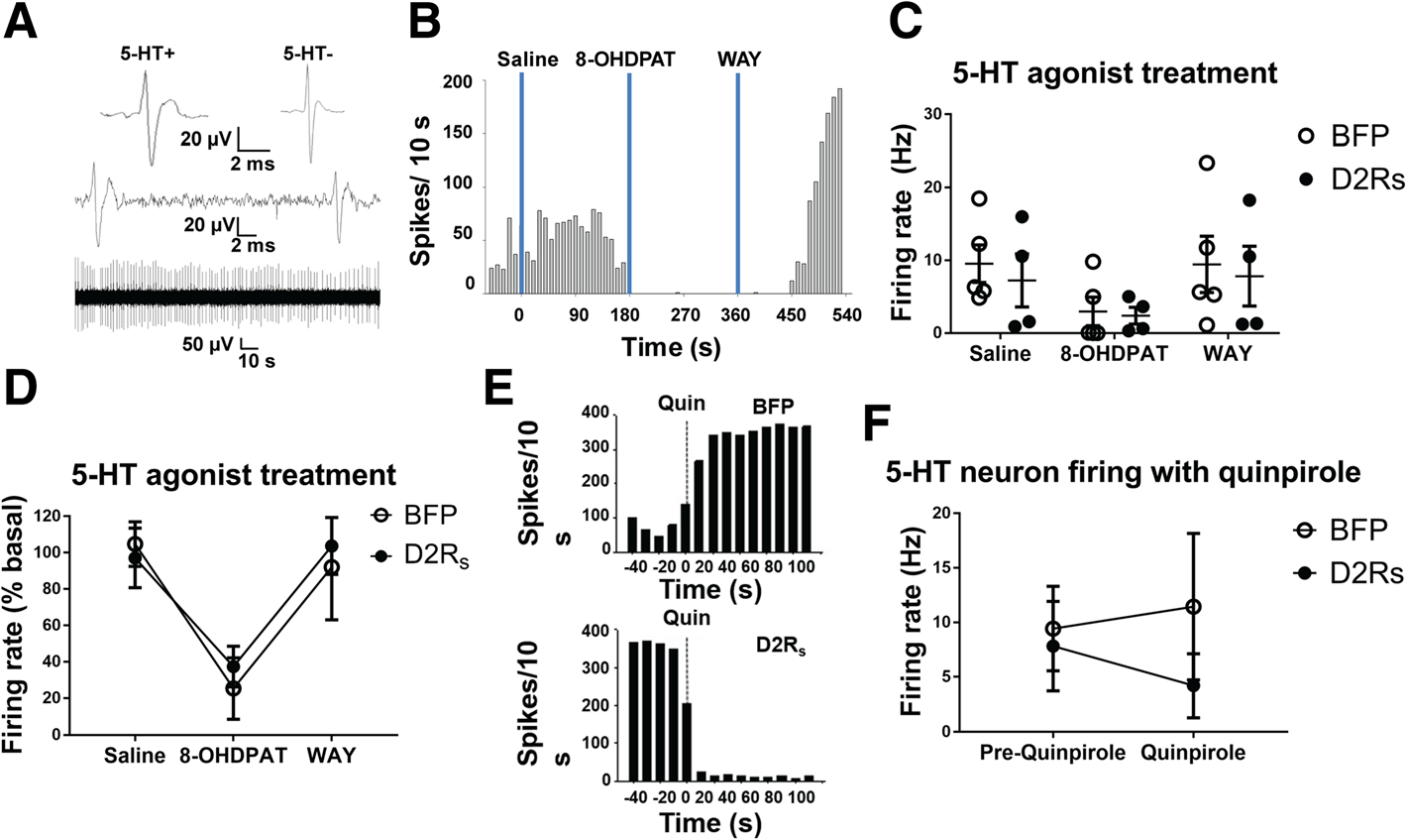
Ectopic DRN D2R_s_ expression reduces 5HT neuronal firing. **a** Top: Traces show typical single-unit recordings of isolated DRN 5-HT neurons (5-HT+) (left) and non-serotonergic (5-HT-) neurons (right). Middle/Bottom: 5-HT neurons often exhibit burst firing with short inter-spike intervals as well as regular spiking. **b** Systemic administration of the selective 5-HT1A agonist 8-OH-DPAT (1 μg/kg, i.v.), but not saline (0.9%) vehicle, suppressed the spontaneous firing of a DRN neuron exhibiting spike characteristic of a 5-HT cell. A return to baseline firing was observed after the local application of WAY-100635 ((100 μg/kg, i.v.), vertical blue bars). **c**-**d** firing rate distributions of DRN neurons recorded in BFP and D2R_s_ expressing rats before and after the application of saline, 8-OH-DPAT and WAY-100635. Putative 5-HT neurons in both groups exhibited similar inhibitory responses to 5-HT1AR agonist and reversal of inhibition by 5-HT1AR antagonism. **e** Firing rate histograms showing the effects of the D2R agonist quinpirole (500 μg/kg, i.v.) on 5-HT neurons recorded in BFP (top) or AAV-D2R_s_ (bottom) injected rats. Control neurons that responded to Quin increased their firing activity to varying degrees, whereas the majority of 5-HT cells recorded in AAV-D2R_s_ injected rats were inhibited. **f** Cumulative electrophysiological data showing the mean ± S.E.M. firing rates of 5-HT DRN cells transfected with BFP or D2R_s_ prior to, and after Quin administration

In order to demonstrate that the ectopically expressed D2R_s_ have the capacity to inhibit the activity of identified 5-HT neurons, we performed electrophysiological recordings on a separate cohort of (intact, non-L-DOPA-treated, non-dyskinetic) animals. Animals received a stereotaxic delivery of either vector as described above, and 4–12 weeks later we performed in vivo single-unit extracellular recordings of DRN neurons. Putative 5-HT neurons were identified based initially on their firing characteristics (e.g., long-duration action potentials, regular firing pattern interrupted with burst activity). Next, neurons were identified as serotonergic based on well characterized responses to systemic administration (i.v.) of 5HT1AR agonist (8-OH-DPAT) and reversal with antagonist (WAY-100635) which restored 5-HT neuron firing to that of baseline (Fig. 6b-d) [15, 33]. Figure 6a shows typical traces of 5-HT and non-5HT DR neurons. Importantly, electrophysiologically identified 5-HT neurons recorded in the dorsal raphe of rats transduced with AAV expressing BFP or D2R_s_ responded similarly to systemic administration of vehicle, 5-HT1AR agonist, and reversal of 5-HT1AR inhibition by 5-HT1AR antagonism. Moreover, 5-HT cells recorded in AAV-D2R_s_ injected rats administered the D2 agonist quinpirole (i.v.) exhibited clear inhibitory effects, whereas responses to quinpirole were variable and sometimes excitatory in BFP controls (Fig. 6e-f). These data show that ectopic expression of D2R_s_ in confirmed 5-HT neurons can act as a functional autoreceptor and inhibit impulse activity in serotonergic neurons.

## Discussion

In this study, we used rAAV to ectopically express the dopamine autoreceptor (D2R_s_) in order to equip DRN 5-HT neurons with a DA-mediated autoregulatory mechanism. Dysregulated DA release from 5-HT neurons through a phenomenon known as “false neurotransmission” has been extensively implicated as a key contributor to LID development [10, 14, 24, 45–47, 54]. While a number of studies have supported this theory, to date, no direct evidence has been presented that shows that DRN neurons can release DA in the striatum and ultimately effect LID. In the present study, our data demonstrate that providing DA-dependent autoregulation in 5-HT neurons can prevent LID formation, thus, providing unambiguous evidence that 5-HT neurons play a central role in DA-dependent symptomology.

Indeed, a wealth of preclinical and clinical studies has shaped the serotonin hypothesis of LID, which suggest that DA synthesis and release from striatal 5-HT terminals is involved in AIM presentation. Specifically, studies ablating DRN neurons or dampening their activity with serotonin autoreceptor agonists have been shown to reduce or eliminate LID; the hypothesized reasoning being that reducing aberrant serotonergic neuronal activity following L-DOPA administration leads to a reduction in striatal DA release from ectopically sprouted DRN terminals [14, 25, 36]. Although the mechanism by which 5-HT neurons process L-DOPA and release DA is not fully established, it is well known that the synthesis and vesicular packaging mechanisms are present in serotonergic neurons [2, 27, 73].

However, while a number of studies have supported this theory, to date, no direct evidence has been provided documenting that DRN neurons are a significant contributor to elevations in striatal DA following L-DOPA; and the role of this mechanism per se in LID expression. In the present study, we demonstrate that when DRN are induced to ectopically express D2R_s_ autoreceptors, 1) hyper-DA release in the striatum following L-DOPA is significantly dampened, presumably by providing DA-dependent autoregulation in striatal 5-HT terminals and 2) that this approach can completely prevent LID formation without compromising motor benefit. These data provide unambiguous evidence that 5-HT neurons play a central role in the DA-dependent pathophysiology of LID.

### Dopamine autoregulation in the dorsal raphe blocks 5-HT neuron activity and LID development

In order to better delineate the role of 5-HT neurons in dyskinesogenesis, we argued that expressing DA regulatory factors in 5-HT neurons would decrease LID severity. 5-HT autoreceptors share a canonical signaling cascade with the D2-type DA autoreceptors—both are inhibitory G-protein coupled receptors (GPCRs) that reduce cellular cAMP to inhibit neuronal signaling [34, 55]. Accordingly, we hypothesized, and have now confirmed, that ectopically expressing the DA autoreceptor D2R_s_ in DRN neurons can serve a physiological autoregulatory function. In support of this, recent work demonstrated that D2R_s_ autoreceptor expression in the DRN of naïve mice results in a reduction of 5-HT-mediated currents [27]. Indeed, in the current study we found using direct recordings of single 5-HT neurons in the DRN that ectopic D2R_s_ expression can provide an inhibitory neuromodulatory effect in 5-HT neurons, characterized by a strong decrease in spontaneous firing following systemic DA D2R agonist administration. Accordingly, we utilized rAAV targeted to the DRN in hemiparkinsonian rats that subsequently received a LID-inducing dosing regimen of L-DOPA. As hypothesized, we found that DRN expression of D2R_s_ provided complete protection against the development of LID, an effect that also persisted at high doses of L-DOPA. Importantly, there was no difference in the extent of nigrostriatal denervation between the groups, nor was there any demonstrable toxicity due to either treatment in the DRN. Thus, prevention of LID was explicitly due to expression of D2R_s_ in the DRN.

### Dopamine efflux into the striatum is reduced with dorsal raphe D2R_s_ expression

Although there is a wealth of research supporting the abnormal serotonergic input in LID development [3, 56, 69], direct evidence showing that the contribution is due to an increase in DA release from these neurons is limited. Using in vivo microdialysis, we have provided the first evidence that L-DOPA mediated DA efflux into the striatum can be significantly modulated by negatively regulating DRN serotonin neurons with D2R_s_ expression. It is notable that we observed a total blockade of LID development with a partial reduction in DA efflux in the striatum. This indicates that a complete block of DA signaling in the striatum is not required for LID inhibition, but rather, mitigation of the pulsatile DAergic tone that occurs with oral administration of L-DOPA is required. Additionally, achieving a total depletion of DA release in the striatum would likely result in a loss of L-DOPA efficacy, as the primary source of L-DOPA metabolism and DA release in severely DA denervated animals originates from DRN neurons. Our data suggests that partial DA efflux reduction and proper DAergic regulation is sufficient to ameliorate LID in our animal model.

In contrast to DA efflux, there was no evidence of decreased 5-HT efflux in the striatum in rAAV-D2R_s_ animals, suggesting 5-HT release was not affected. This is surprising given our finding that autoreceptor stimulation effectively reduces 5-Ht neuron firing. One likely explanation for this observation is that the lack of impact on striatal 5-HT release was due to a lack of direct stimulation of 5-HT release concomitant with L-DOPA treatment, thus, our measurements reflected baseline 5-HT release. Nevertheless, our findings demonstrate that D2R_s_ can induce DAergic regulation in 5-HT neurons, by ‘hijacking’ endogenous signaling cascades and reducing neuronal activity following L-DOPA administration.

Taken together, our in vivo electrophysiology and microdialysis data suggest that the mechanism by which expression of D2R_s_ in DRN neurons provides complete protection against the development of LID is through a neuromodulatory feedback mechanism. This is further supported based on equal levels of nigral DA neuron loss between rAAV-D2R and rAAV-GFP groups, supporting that this antidyskinetic efficacy was explicitly due to expression of D2R_s_ in the DRN.

Our data indicate that exogenously provided D2R_s_ can couple with G_αi_ subunits in DRN neurons, and induce the appropriate signaling cascades to reduce neuronal activity in the presence of exogenous L-DOPA. In conjunction with the LID studies utilizing serotonin agonists, our data confirm that reducing the activity of the serotonin system can dramatically inhibit LID. However, the critical advantage of this target-specific gene therapy approach over pharmacological therapy [19, 36, 37, 58] is that there is no decrease in motor benefit of L-DOPA. While this is the first evidence showing that serotonergic neurons, when supplied exogenously with a single DA-regulatory factor, can modulate DA release and completely prevent the induction of LID in a ‘prevention’ scenario, future studies aimed at examining the capacity of this mechanism to reduce or reverse established LID will be imperative.

### Ectopic D2R_s_ expression in the dorsal raphe blocks L-DOPA priming in the striatum

In order to better understand the global impact of striatal DA regulation via DRN D2R_s_ expression on an array of DA therapies in parkinsonian subjects, we tested the hypothesis that the protective effects of this autoreceptor treatment would be negated in the presence of DA-receptor agonists which directly bind to DA receptors on striatal medium spiny neurons (MSNs). We reasoned that since the DA regulation thru the D2R_s_ is a presynaptic mechanism, that treatment with DA receptor agonists, which act at postsynaptic receptors that become supersensitive with striatal DA depletion and result in dyskinesias in animal models and patients [11, 12, 20, 31]—should induce AIMs in rAAV-D2R_s_-treated animals resistant to LID. To our surprise, treatment with D1-, D2-specific, or pan-DA agonists did not induce severe AIMs in rAAV-D2R_s_ animals, and only a mild-to-modest dyskinetic response was seen with the D1 agonist SKF-81297, the last of the three DA agonist drugs tested. This would suggest that D2R_s_ therapy disallowed LID priming to occur in striatal MSNs. The autoreceptor allows for proper regulation of DA signaling from DRN neurons, removing the pulsatile stimulation induced by intermittent DA dosing which is important in LID development. Thus, the MSNs of rAAV-D2R_s_ treated animals first exposure to abnormal DA signaling would be at the initial agonist challenge, where priming could begin. Accordingly, this increase in AIMs behavior with the D1 agonist may have been due to a mild degree of DA-agonist induced priming, a phenomenon that is to be expected as direct MSN DA receptor activation would not be mitigated by DRN D2R_s_ expression. This is supported by the experimentation by Carta and colleagues, where the co-administration of apomorphine with the 5-HT_1A_ agonist *after* an induction period where L-DOPA was administered over 3 weeks, did not alleviate LID, suggesting that the induction phase irreversibly primed the neurons to LID [14].

It is well established that LID development is associated with a “priming-period” consisting of discontinuous, non-physiological, striatal DA tone that results in morphological and molecular changes to the MSNs [13, 16, 53, 60, 70, 72, 78]. Our data therefore indicates that D2R_s_-treated animals were blocked from the L-DOPA priming by counteracting the non-physiological surges of DA release, thereby preventing a host of pathological molecular mechanisms that may include normalizing post-synaptic striatal DA receptor supersensitivity. The fact that at the end of the treatment we began to observe a mild-to-moderate increase in AIM presentation in rAAV-D2R_s_ animals with DA agonist treatment as compared to L-DOPA, suggest that these animals were in the early stages of priming, a phenomenon that is to be expected as direct MSN DA receptor activation would not be mitigated by DRN D2R_s_ expression. Future studies examining the molecular mechanisms associated with prevention of L-DOPA- and DA agonist-induced priming, and the durability of this prevention with DA agonist therapy in particular are warranted. While there was a break between L-DOPA and DA agonist treatment (Fig. 1a) this would not affect the primed state or future maintenance of LID, as this type of ‘drug holiday’ does not ameliorate LID when a patient or animal model is reintroduced to a DAergic therapy [74, 75]. Future studies challenging L-DOPA naïve rAAV-D2R_s_ rats with DA agonists, and altering the order of agonist treatment, would allow us to determine the role of priming and sensitization with this treatment.

### Inhibition of dorsal raphe serotonergic neurons does not mitigate the anti-parkinsonian benefits of L-DOPA

As briefly discussed above, it was important to confirm that D2R_s_ expression in the DRN does not negatively affect the therapeutic efficacy of L-DOPA in our PD model, as this has been an issue with serotonin agonist-type therapies in clinical trials for LID [19, 37, 58], and an imperative problem to mitigate for all future therapies. The current studies demonstrate that this gene therapy approach of providing DA autoregulatory properties to DRN neurons results in no changes in motor improvement between control and D2R_s_ animals. This was confirmed in two separate cohorts of rats and using two different motor tests. Both tests demonstrated that rats with the D2R_s_ in DRN neurons maintain a significant improvement in motor function with the administration of L-DOPA, reflecting recovery back to a pre-lesion state. This shows that D2R_s_ activity in serotonergic terminals of the striatum (or elsewhere) does not interfere with the pharmacological benefits of L-DOPA, and implicates D2R_s_ therapy as a potential potent treatment option for LID. It is important to note that while many preclinical studies using 5-HT agonists did not show an effect on L-DOPA-induced motor improvement, these results have not translated clinically. While multiple trials have used a variety of 5-HT agonists and seen reductions in AIM scores, many of these compounds contribute to worsening of parkinsonian symptoms and OFF L-DOPA periods, or have been abandoned due to lack of antidyskinetic efficacy (reviewed in [19]). The discrepancy between our D2R_s_ approach and the use of agonists is unclear given that these two approaches conceivably evoke the same mechanism. Nevertheless, 5-HT1 compounds may produce their own side effects [43]. Second, their effects are dependent on an exogenously administered compound and hold a potential for suboptimal dosing (and timing of administration) as opposed to a gene therapy approach. Nevertheless, further studies are warranted to determine if D2R_s_ expression in the raphe is successful in other preclinical models of LID.

Pharmacological manipulations of 5-HT neurons in the treatment of LID, although successful pre-clinically, have not been fully translated. The transient nature of the anti-dyskinetic effect of currently available 5-HT approaches may be due to pharmacologic limitations of these drugs, including lack of specificity and potency for the specific receptor. Moreover, timing and comparative pharmacodynamics with L-DOPA delivery may be preventative [51]. Because of this, a genetic approach in the form of continuous 5-HT inhibition should bypass such pharmacological limitations and provide meaningful and lasting protection against LID. Moreover, the finding that D2R_s_ gene therapy does not interfere with L-DOPA efficacy in our rat model provides promise for such an approach. Of course, the DR innervates a large part of the brain, providing many crucial functions, and the D2R_s_ therapy undertaken here does not distinguish between various projections. Thus, understanding any off-target effects from DA-mediated regulation of 5-HT neurons remains one important caveat that requires further research. Further studies to better understand potential side effects and the effect on the serotonergic system may be warranted. Moreover, it is important to point out that our study is limited in that we provided no genetic precision with our vector delivery as would be afforded in, for instance, a CRE animal. Although we observed a majority of somatic transduction in the area of the DR, it is also possible that other circuits were transduced with our vectors. Accordingly, future efforts should be aimed at limiting transgene expression to DR 5-HT neurons.

Our findings bring to light previous work demonstrating changes in 5-HT innervation occurring concomitant with nigrostriatal denervation and PD. Both 5-HT hyperinnervation [4, 62, 65] as well as a decrease in 5-HT terminals [32, 39, 40, 67] has been documented in human disease. Although the cause of these divergent findings is unknown, it is highly likely that 5-HT neurons play an important role in PD symptomology and, as our findings would suggest, in LID. As nigrostriatal denervation in human PD is near complete at the time of diagnosis [41] it is conceivable to speculate that changes in 5-HT innervation and function—and the capacity of these neurons to release DA—is a crucial component to dyskinesogenesis. To that end, understanding both the mechanisms of how 5-HT neurons process and release DA, and the underlying etiology of presynaptic 5-HT changes are important components as we begin to understand LID etiology and PD nonmotor symptoms, and represents a new therapeutic modality.

## Conclusions

In conclusion, the current study shows that DA release from DRN 5-HT neurons can be regulated with ectopic expression of D2R_s_, altering the activity and DA release properties of these neurons in a therapeutically meaningful way. These data add important evidence to the current understanding of LID and serve as to confirm the serotonin hypothesis in LID, showing that directly regulating serotonin neuron activity can inhibit LID development.

## Additional files

Additional file 1: **Figure S1.** Concentrations of Monoamines in Lesioned vs. Intact side of brain. Concentration (picograms per microliter) of monoamines and metabolites for striatal tissue taken from animals used in microdialysis experiments. We observed a drastic reduction in DA (>~ 98% of intact hemisphere) and DOPAC (>~ 89% of intact hemisphere) levels in the lesioned striatum of either vector group, indicating successful lesions. There were no significant differences in any monoamine concentrations in GFP vs rAAV-D2R rats. (TIF 8243 kb)

Additional file 2: **Figure S2.** Evaluation of transgene expression and effect on DRN neurons. (A and B) IHC for virally expressed transgenes D2R_s_ (A) or GFP (B) show substantial expression throughout brain. The widespread immunoreactivity indicates DRN innervation targets. Transgene was observed in striatal projection fibers from the DRN (C, G). No cell bodies were transduced in regions outside of the raphe, including the SNc (D, H). IBA1 immunoreactivity showed a slight microgliosis at the injection site in both vector groups (E, I) but not elsewhere. 5-HT immunoreactivity was comparable between groups (F, J) and the number of 5-HT+ DRN neurons was the same in both groups (L). Scale bars: A, B = 1 mm; C, D, G, H = 50 μm; E, F, I, J = 100 μm. Boxes in A and B outlines areas of magnification in C, D and G, H respectively. (TIF 27770 kb)

Additional file 3: **Figure S3.** AIM scores in L-DOPA dosing paradigm. AIM scores for days 1–10 in the L-DOPA dosing regimen, ranging from 2 mg/kg-8 mg/kg. Significantly more severe AIMs were observed in rAAV-GFP animals starting on day 10 with 8 mg/kg. Peak-dose severity scores taken at 75 min post L-DOPA. (* = *p* ≤ 0.05, ** = *p* ≤ 0.01, *** = *p* ≤ 0.001). (TIF 52816 kb)
