# Supplementary figures and images for: Regulation of dopamine neurotransmission from serotonergic neurons by ectopic expression of the dopamine D2 autoreceptor blocks levodopa-induced dyskinesia
Source: Acta Neuropathol Commun. 2019 Jan 15;7:8. doi: 10.1186/s40478-018-0653-7 (PMC6332643; doi:10.1186/s40478-018-0653-7)

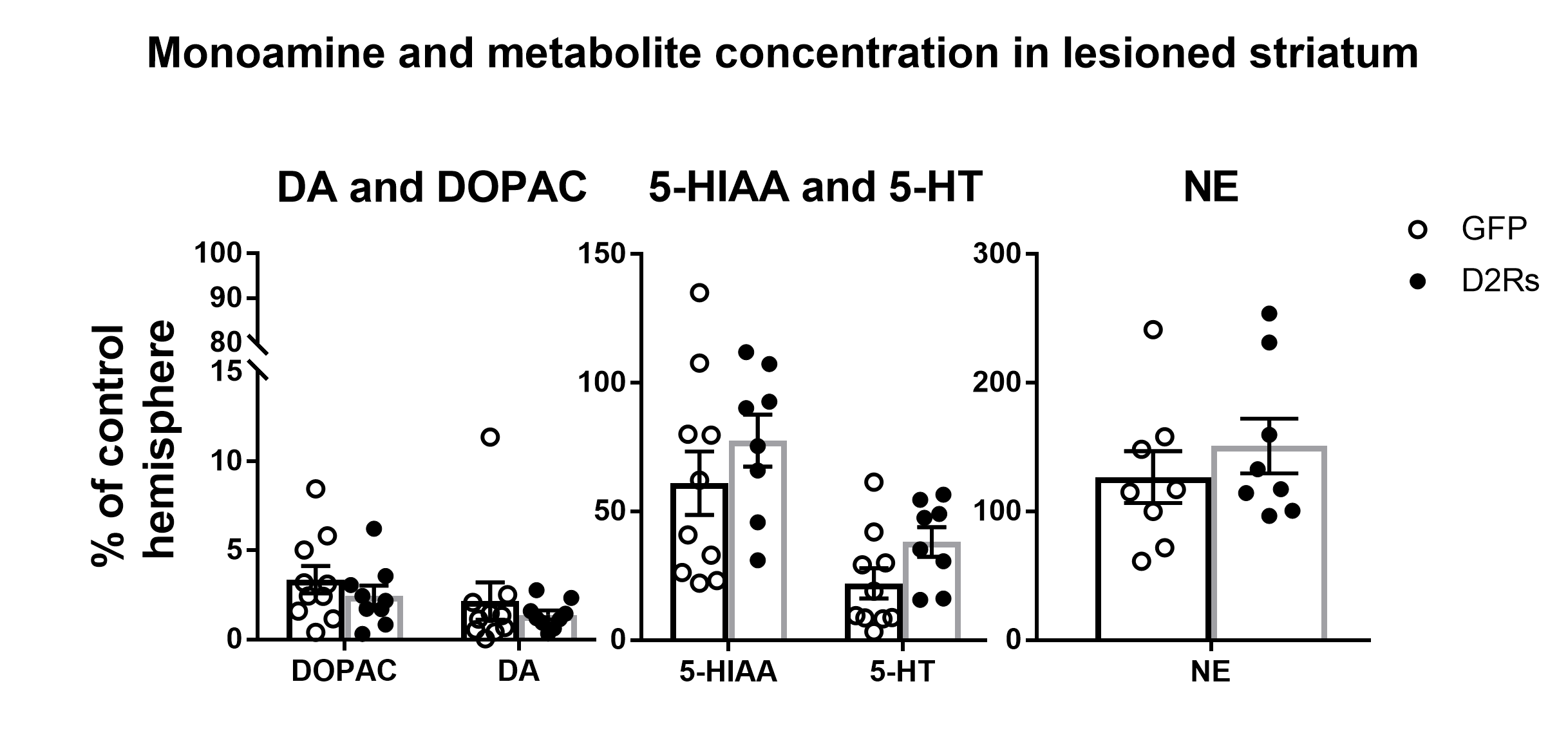

Supplement: Supplementary file 1 — Figure S1. Concentrations of Monoamines in Lesioned vs. Intact side of brain. Concentration (picograms per microliter) of monoamines and metabolites for striatal tissue taken from animals used in microdialysis experiments. We observed a drastic reduction in DA (>~ 98% of intact hemisphere) and DOPAC (>~ 89% of intact hemisphere) levels in the lesioned striatum of either vector group, indicating successful lesions. There were no significant differences in any monoamine concentrations in GFP vs rAAV-D2R rats. (TIF 8243 kb) [file 40478_2018_653_MOESM1_ESM.tif]

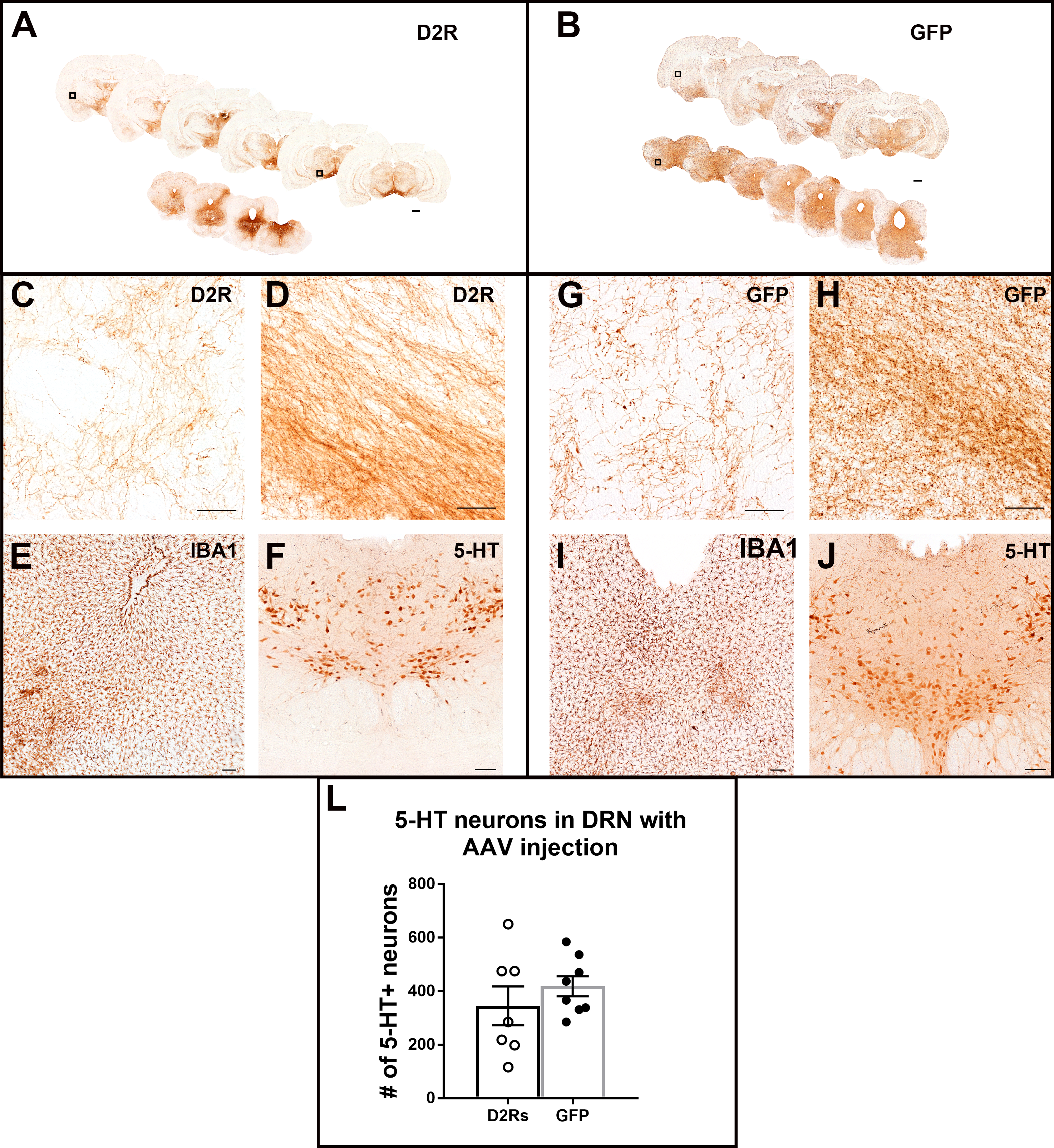

Supplement: Supplementary file 2 — Figure S2. Evaluation of transgene expression and effect on DRN neurons. (A and B) IHC for virally expressed transgenes D2Rs (A) or GFP (B) show substantial expression throughout brain. The widespread immunoreactivity indicates DRN innervation targets. Transgene was observed in striatal projection fibers from the DRN (C, G). No cell bodies were transduced in regions outside of the raphe, including the SNc (D, H). IBA1 immunoreactivity showed a slight microgliosis at the injection site in both vector groups (E, I) but not elsewhere. 5-HT immunoreactivity was comparable between groups (F, J) and the number of 5-HT+ DRN neurons was the same in both groups (L). Scale bars: A, B = 1 mm; C, D, G, H = 50 μm; E, F, I, J = 100 μm. Boxes in A and B outlines areas of magnification in C, D and G, H respectively. (TIF 27770 kb) [file 40478_2018_653_MOESM2_ESM.tif]

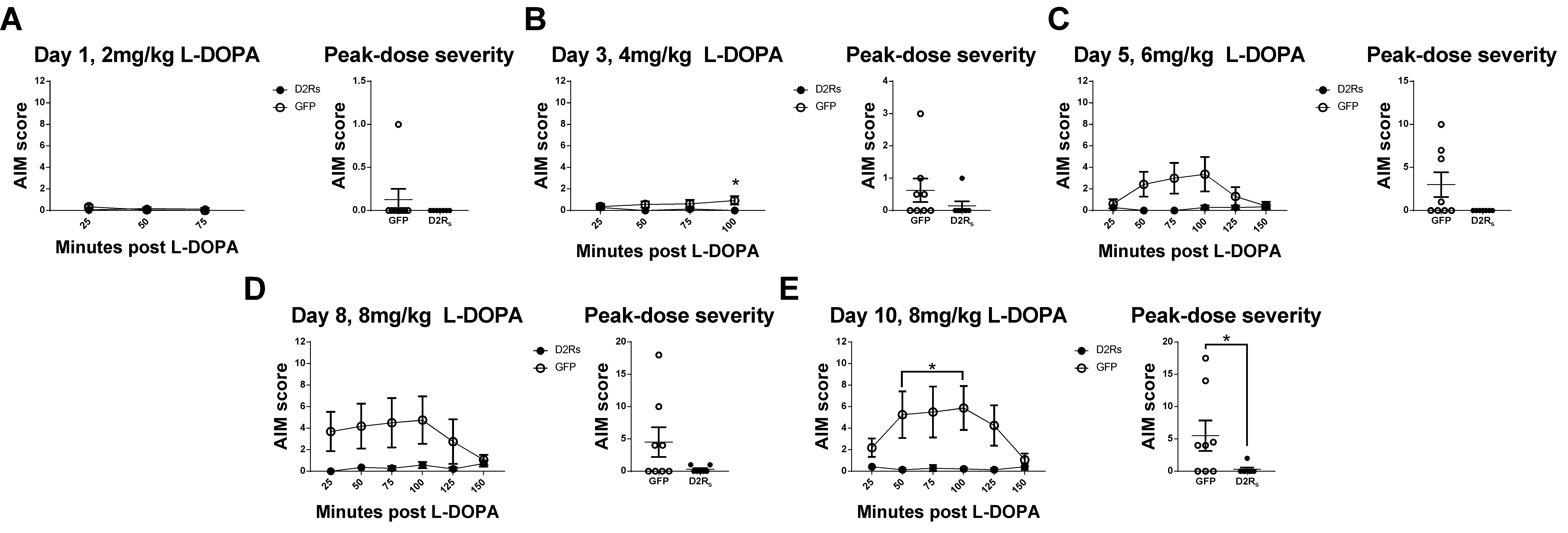

Supplement: Supplementary file 3 — Figure S3. AIM scores in L-DOPA dosing paradigm. AIM scores for days 1–10 in the L-DOPA dosing regimen, ranging from 2 mg/kg-8 mg/kg. Significantly more severe AIMs were observed in rAAV-GFP animals starting on day 10 with 8 mg/kg. Peak-dose severity scores taken at 75 min post L-DOPA. (* = p ≤ 0.05, ** = p ≤ 0.01, *** = p ≤ 0.001). (TIF 52816 kb) [file 40478_2018_653_MOESM3_ESM.tif]
